# An Innovative Approach to a Potential Neuroprotective *Sideritis scardica*-Clinoptilolite Phyto-Nanocarrier: In Vitro Investigation and Evaluation

**DOI:** 10.3390/ijms25031712

**Published:** 2024-01-30

**Authors:** Adina-Elena Segneanu, Gabriela Vlase, Titus Vlase, Andrei Bita, Cornelia Bejenaru, Gabriela Buema, Ludovic Everard Bejenaru, Andrei Dumitru, Eugen Radu Boia

**Affiliations:** 1 Institute for Advanced Environmental Research-West University of Timisoara (ICAM-WUT), Oituz nr.4, 300223 Timisoara, Romania; gabriela.vlase@e-uvt.ro (G.V.); titus.vlase@e-uvt.ro (T.V.); 2 Research Centre “Thermal Anal Environm Problems”, Institute for Advanced Environmental Research-West University of Timisoara (WUT), Pestalozzi St 16, 300115 Timisoara, Romania; 3 Department of Pharmacognosy & Phytotherapy, Faculty of Pharmacy, University of Medicine and Pharmacy Craiova, St. Petru Rareș 2, 200349 Craiova, Romania; andrei.bita@umfcv.ro (A.B.);; 4 Department of Pharmaceutical Botany, Faculty of Pharmacy, University of Medicine and Pharmacy Craiova, St. Petru Rareș 2, 200349 Craiova, Romania; cornelia.bejenaru@umfcv.ro; 5 National Institute of Research and Development for Technical Physics, 47 Mangeron Boulevard, 700050 Iasi, Romania; gbuema@phys-iasi.ro; 6 Faculty of Sciences, Physical Education and Informatics—Department of Medical Assistance and Physiotherapy, National University for Science and Technology Politehnica Bucharest, University Center of Pitesti, Targu din Vale 1, 110040 Pitesti, Romania; andrei.dumitru@upit.ro; 7 Department of Ear, Nose, and Throat, Faculty of Medicine, “Victor Babeș” University of Medicine and Pharmacy Timisoara, 2 Eftimie Murgu, 300041 Timisoara, Romania; eugen.boia@umft.ro

**Keywords:** ironwort, zeolite, phytochemicals, drug delivery systems, antioxidant potential, dissolution profile

## Abstract

The cutting-edge field of nanomedicine combines the power of medicinal plants with nanotechnology to create advanced scaffolds that boast improved bioavailability, biodistribution, and controlled release. In an innovative approach to performant herb nanoproducts, *Sideritis scardica Griseb* and clinoptilolite were used to benefit from the combined action of both components and enhance the phytochemical’s bioavailability, controlled intake, and targeted release. A range of analytical methods, such as SEM-EDX, FT-IR, DLS, and XDR, was employed to examine the morpho-structural features of the nanoproducts. Additionally, thermal stability, antioxidant screening, and in vitro release were investigated. Chemical screening of *Sideritis scardica Griseb* revealed that it contains a total of ninety-one phytoconstituents from ten chemical categories, including terpenoids, flavonoids, amino acids, phenylethanoid glycosides, phenolic acids, fatty acids, iridoids, sterols, nucleosides, and miscellaneous. The study findings suggest the potential applications as a promising aspirant in neurodegenerative strategy.

## 1. Introduction

*Sideritis scardica Griseb* (*Lamiaceae* family) has been known for its medicinal properties since ancient times. Renowned scholars and physicians such as Hippocrates, Discorides, Theophrastus, and Galen have recommended this plant for wound regeneration and healing caused by iron weapons. In Southeast Europe and Turkey, the plant is used for flavoring or preparing tea, hence its common name, ironwort or “*Mountain tea*” [1,2]. In addition, there are other local names for this plant. For instance, in Bulgaria, it is known as “*Pirin tea*”, “*Mursalski tea*”; or “*Alibotushki tea*”. In Greece, it is referred to as “Greek Mountain tea” or “Olympus tea”, while in North Macedonia, it is called “*Sharplaninsi chaj*” [1,2,3]. *Sideritis scardica* is highly valued in traditional medicine in these regions and is considered one of the most famous herbs, known for its astonishing therapeutic properties such as antioxidant, anti-inflammatory, anti-ulcerogenic, digestive, and antimicrobial effects. Recent research reported the notable biological activity (antiseptic, diaphoretic, anti-rheumatic, gastroprotective, antidiabetic, antiproliferative, anti-HIV, and neuroprotective) because of the collective action of its highly complex phytoconstituents composition [1,3,4,5].

Current studies have focused on the development of nanomaterials development derived from natural compounds to overcome the in vivo limitations of some secondary metabolites. These limitations may include chemical or thermal instability, poor bioavailability, limited membrane phytoconstituents transport, and others. Through the creation of nanomaterials, prolonged action, vectorization, specificity, and superior therapeutic action can be achieved [6,7,8,9,10].

Clinoptilolite-zeolite is a widely available, low-cost, crystalline hydrated aluminosilicate with a unique structure, regular microporous, high thermal and chemical stability, and high ion exchange selectivity. Due to their extraordinary morpho-structural properties, clinoptilolite has proven to be a highly versatile material with numerous potential applications across various fields, including agriculture, industry, catalysts, environmental protection, and biomedicine. Studies have also shown that this mineral can have several beneficial effects on human health, such as anti-inflammatory, antioxidant, antibacterial, antitumoral immunomodulating agent, and protecting against neurodegenerative diseases [11,12,13,14,15,16,17]. Recent research has demonstrated their efficacy in various medical applications (biosensors, diagnosis, drug delivery systems, bone tissue engineering, detoxification, dental treatments, and more) [11,12,13,14,15,16,17]. Although clinoptilolite’s possible side effects were controversial issues concerning therapeutic uses, the latest research reported that micronized eliminates these shortcomings and increases the efficiency of biomedical applications [12,13]. Numerous literature results report that clinoptilolite exhibits remarkable potential as a carrier platform, owing to its ability to significantly enhance both the shell life and biocompatibility of phytoconstituents in vivo, thereby facilitating controlled and prolonged release [11,13,15,18,19,20]. Specifically, the unique physicochemical properties of clinoptilolite have been shown to effectively stabilize and protect phytoconstituents from degradation and toxicity while also improving their bioavailability and therapeutic efficacy [11,13,15,18,19,20]. These findings suggest that clinoptilolite represents a promising platform for the development of advanced drug delivery systems that can overcome many of the limitations associated with conventional drug delivery methods [11,19,20].

Nevertheless, as the importance of safe and effective self-medication increases worldwide, the need to explore alternatives to traditional drugs is becoming more pressing. Medicinal plant preparations offer a compelling opportunity, providing beneficial health effects, treating ailments, and boosting immunity through their potent antioxidant, antimicrobial, antiviral, anti-inflammatory, and antitumor properties. However, research has indicated that self-administration of herbal products can result in exceeding optimal doses, leading to severe complications [21,22,23,24,25,26]. Therefore, the highly effective herb product development that addresses these concerns by providing controlled intake and release of biomolecules, increasing bioavailability, and minimizing toxicity is paramount. Accordingly, herbal products can offer a safe and effective solution to the problem of antibiotic resistance and harmful side effects associated with traditional drugs.

To this end, this study uses a novel, simple approach for developing a new carrier system that combines ironwort and clinoptilolite to harness their collective action. The physicochemical properties, antioxidant potential, and in vitro dissolution test of this new nanocarrier were investigated thoroughly.

## 2. Results and Discussion

Various studies focused on the chemical screening and biological activity of *Sideritis scardica*, one of the most famous medicinal plants in the Balkan region. Nevertheless, variation in the chemical composition of a plant depending on numerous biotic, abiotic, and experimental parameters greatly complicates the establishment of an interdependence between the biological activity of a medicinal plant and the profile of secondary metabolites [27,28].

Numerous studies have reported that several phytoconstituents with outstanding biological activity exhibit in vivo reduced adsorption and reduced permeability, corresponding to their size, solubility, and stability (thermal and chemical). The limited target specificity, transmembrane permeability, and retention of these compounds also represent significant obstacles to current therapeutic strategies for severe diseases [28,29,30,31].

Nonetheless, identifying and addressing these challenges will lead to the development of effective treatments for severe diseases [31]. Therefore, innovative approaches are imposed to overcome these challenges.

Conversely, certain phytoconstituents isolation and then their total chemical synthesis or semi-synthesis are essential and challenging steps related to the complex chemical structure (stereochemical centers, and others) [27,28,32,33]. Nevertheless, the latest literature results have shown that the combined therapeutic effects of all secondary metabolites in a whole plant are superior to the biological activity of a single biomolecule [27,28,32,33]. Accordingly, the design of phytocarrier systems that combine the biological action of all secondary metabolites from a medicinal plant and that of the carrier can overcome the current therapeutic strategy limitations and allow controlled release and target specificity.

Complete screening of low-weight phytoconstituents from ironwort sample was performed using gas-chromatography coupled with mass spectroscopy (GC-MS) and electrospray ionization–quadrupole time-of-flight mass spectrometry (ESI-QTOF-MS) techniques.

Gas chromatography coupled with mass spectrometry (GC-MS) is one of the most common, fast, and easy techniques for the specific identification of volatile molecules from a complex mixture [34].

However, non-volatile and or thermolabile metabolites require additional methods of protection by chemical derivatization before GC-MS analysis [35].

### 2.1. Screening of Amino Acid from Sideritis Scardica Sample

**Figure 1:**
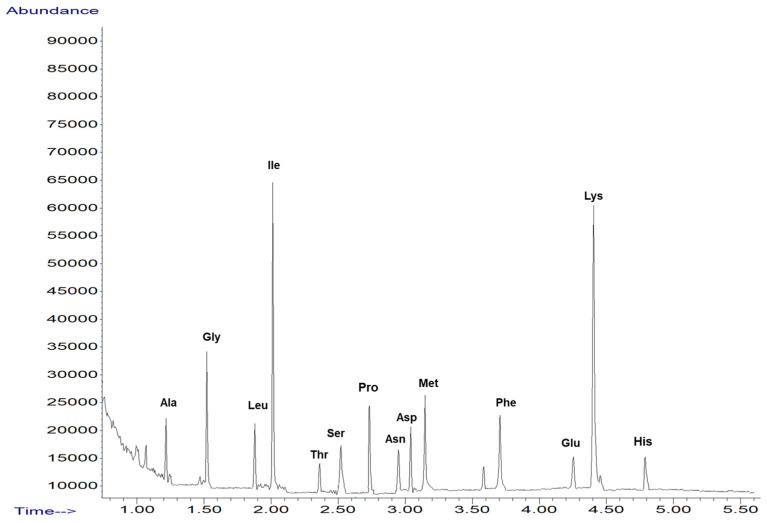
TIC chromatograms of GC-MS for *Sidertis scardica*.

The amino acid profile from the ironwort sample was investigated using GC-MS techniques (Figure 1). The compounds were identified based on the retention indices, mass spectra with those of NIST/EPA/NIH, the Mass Spectral Library 2.0 database, and the literature [36,37,38].

Table 1 presents the tentative amino acid identification via GC-MS corresponding to the *Sideritis scardica* sample.

### 2.2. Mass Spectrometry Analysis of Sideritis Scardica Sample

The MS results depicted a highly heterogenous composition of compounds, of which some were detected, as pertaining to several chemical categories (amino acids, terpenoids, iridoids, phenylethanoid glycosides, sterols, fatty acids, flavonoids, phenolic acids, and others) that confirmed the data reported in the literature [37,39,40,41,42,43,44,45,46,47,48,49,50].

**Figure 2:**
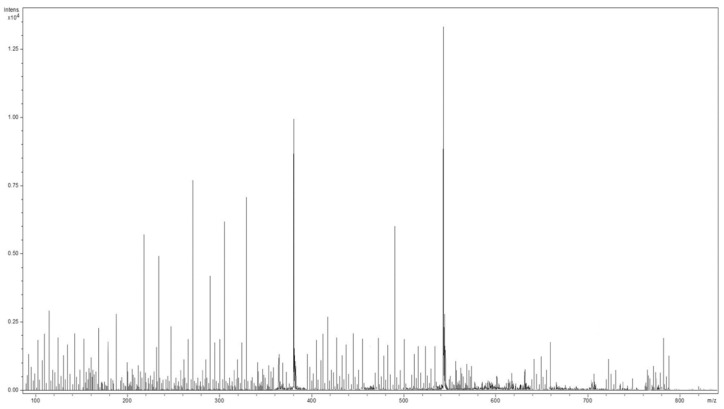
The mass spectra of *Sideritis scardica*.

The mass spectrum and the biomolecules identified via ESI–QTOF–MS analysis are shown in Figure 2 and Table 2, respectively.

### 2.3. Screening and Classification of the Differential Metabolites

A total of 91 secondary phytochemicals identified through mass spectroscopy were assigned to different chemical classes: terpenoids (24.18%), flavonoids (12.08%), amino acids (14.28%), phenylethanoid glycosides (8.8%), phenolic acids (8.8%), fatty acids (5.5%), iridoids (3.3%), sterols (3.3%), nucleosides (2.2%) and miscellaneous.

**Figure 3:**
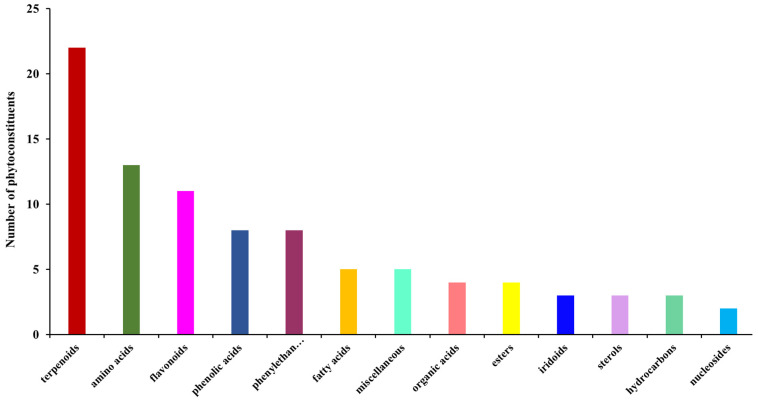
Phytochemical classification bar chart for *Sideritis scardica*.

Figure 3 presents the classification chart of the phytoconstituents from the *Sideritis scardica* sample based on the data analysis reported in Table 2.

According to Figure 3, terpenoids represent the biggest class of secondary metabolites, comprising about 24.18%. Various research reported their remarkable biological properties: anti-inflammatory, antitumor, analgesic, antimicrobial, antiviral, neuroprotective, antispasmodic, antihyperglycemic, antiplasmodial, cardioprotective, and immunomodulatory [52].

Flavonoids are a category of metabolites with noteworthy therapeutic activity: antioxidant, anti-inflammatory, cardioprotective, neuroprotective, antimicrobial, antiviral, and antitumoral [53,54].

Amino acids play a crucial role in maintaining the overall human health. In the ironwork sample, a total of eleven compounds were identified, with essential and non-essential amino acids present in almost equal proportions. The therapeutic properties of amino acids have been extensively studied in biochemistry. Recent research has shown that a significant portion of the amino acids identified in the ironwort sample (over 46% of all amino acids identified) such as isoleucine, glycine, alanine, histidine, phenylalanine, and glutamic acid, exhibit antiproliferative, cytotoxic, and immunomodulating activities [55,56,57,58]. Additionally, amino acids like methionine, proline serine, lysine, threonine, and asparagine have been found to possess anti-inflammatory properties [59,60,61,62,63].

In addition, lysine exerts neuroprotection activity and serine cytoprotective and antiatherogenic effects [64,65].

*Phenylethanoid glycosides* (PhGs) are metabolites with notable biological activities (antioxidant, antibacterial and neuroprotective) [66].

*Phenolic acids* represent 9% of the phytoconstituents from the ironwort sample. Numerous research have demonstrated their pharmacological activity (antioxidant, antimicrobial, cardioprotective, anti-inflammatory, neuroprotective, antitumor, and antidiabetic) [67].

*Iridoids* act *as* neuroprotective, hepatoprotective, anti-inflammatory, antitumor, hypoglycaemic, and hypolipidemic agents [68].

*Nucleosides* uridine and guanisidine act as neuroprotective, neuromodulator, and neurodegenerative agents [69,70].

*Fatty acids* represent 5.5% of the metabolites from the ironwort sample. Recent studies reported their cardioprotective, anti-inflammatory, antioxidant, and neuroprotective effects [71,72].

*Phytosterols* in the proportion of about 3.4% from biomolecule categories identified in ironwort samples have demonstrated antioxidant, anti-inflammatory, neuroprotective, immunomodulatory, and antitumoral [73].

### 2.4. Phyto-Nanocarrier System

Numerous studies have reported that several phytoconstituents with outstanding biological activity exhibit in vivo reduced adsorption and reduced permeability because of their size, solubility, and stability (thermal and chemical) [74].

The limited target specificity, transmembrane permeability, and retention of these compounds also represent significant obstacles to current therapeutic strategies for severe diseases. Nonetheless, identifying and addressing these challenges will lead to the development of effective treatments for severe diseases. Therefore, innovative approaches are imposed to overcome these challenges [31,75,76]. Consequently, the development of an advanced phyto-nanocarrier system by loading secondary metabolites from *Sideritis scardica* into clinoptilolite particles combines the biological activity of the phytoconstituents and the carrier enhancing the therapeutical performance (pharmacological effects, selectivity, controlled released, and bioavailability).

#### 2.4.1. FT-IR Spectroscopy

Fourier transform infrared (FT-IR) spectroscopy is a simple, fast, and efficient technique for compound identification from complex materials [77].

**Figure 4:**
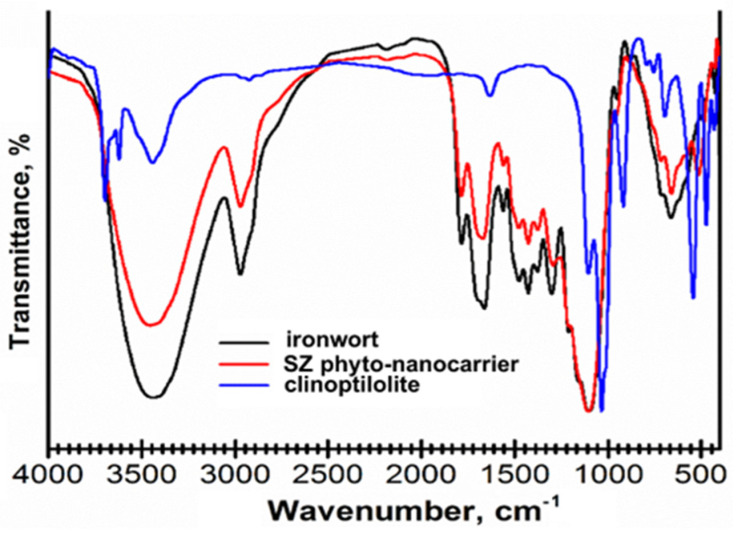
FT-IR spectra of ironwort, clinoptilolite, and SZ phyto-nanocarrier system.

SZ phyto-nanocarrier preparation was investigated using FT-IR spectroscopy to identify the presence of functional groups of both components (herb and clinoptilolite) (Table 3). FT-IR spectra of each constituent are shown in Figure 4.

FT-IR spectrum of clinoptilolite (Figure 4) displays the vibrational bands characteristic at 3630 cm^−1^ (attributed to Si–O(H)–Al), 1645 cm^−1^ (H-O-H deformation vibration), 1230 cm^−1^ (associated with Si (Al)-O asymmetric stretching vibration) 1060 and 795 cm^−1^ (associated to Si-O stretching vibration), 605 cm^−1^ (corresponding to Si-O-Al and Si-O-Si bending vibrations), 554 cm^−1^ (assigned to Si-O-Si symmetric stretching vibration and O-Si-O bending vibration), and 470 cm^−1^ (assigned to Si-O-Si vibrational deformation) [93,94,95].

The FT-IR spectra of the SZ phyto-nanocarrier (Figure 4) include all the characteristic peaks of ironwort (at 3383 cm^−1^ (-OH group), 2880 (C-H),1745 (cyclopentanone ring of iridoids), 1706 (carboxyl acid C=O stretching), 1630 (COO- stretching vibration), 1644 (N-H of amino acids), 1385 cm^−1^ (C-H bending), 1242 and 1015 (C-N of amine), 880 and 817 cm^−1^ (C-O and CH vibration of aromatic rings), as well the clinoptilolite vibrational bands. However, several changes are detectable in the intensity of bands in the C-O, C-H, and N-H regions (1573, 1382, 1250, 1205 cm^−1^). In addition, the absorption bands characteristic to clinoptilolite at 554 (Si-O-Si) and 795 cm^−1^ (Si-O-Si and O-Si-O) are shifting to lower wavenumbers indicating that this functional group is involved in SZ phyto-nanocarrier formation.

#### 2.4.2. X-ray-Diffraction Spectroscopy

X-ray-diffraction spectroscopy (XRD) is a rapid, routine, cost-effective analytic method used to obtain information on the atomic structure of a material and phase identification [96].

**Figure 5:**
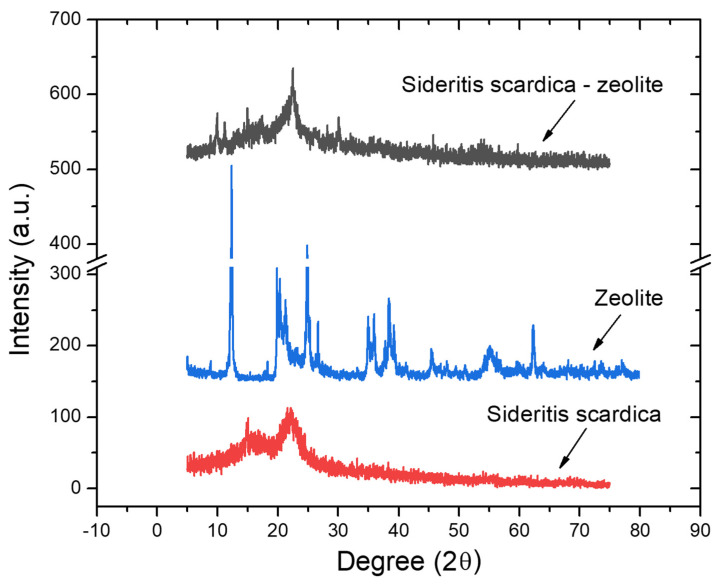
Powder XRD patterns of ironwort, clinoptilolite, and SZ phyto-nanocarrier.

The XRD patterns of SZ phyto-nanocarrier, ironwort, and clinoptilolite are shown in Figure 5.

Clinoptilolite presents diffraction peaks (at 2θ: 11.15°, 17.28°, 22.17°, 22.38°, 23.54°, 24.19°, 26.18°, and 33.39°) of crystalline phase of clinoptilolite zeolite structure with a crystallite mean size of 31.9 nm calculated using Scherrer equation (Figure 5) [97,98].

The corresponding spectrum of ironwort (Figure 5) displays large bands and weak diffraction peaks (in the range of 14.9–31.23°) characteristic of amorphous phases that can be assigned to different minerals and fibers from ironwort [99].

In the XRD pattern of the SZ phyto-nanocarrier are visible the diffraction peaks from both components (clinoptilolite and ironwort) are nonetheless significantly attenuated. Moreover, the characteristics peaks of clinoptilolite (at 2θ: 11.15°, 17.28°, 22.17°, and 22.38°) are shifted to lower angles that suggest the expansion of the zeolite lattice due to the loading of herbs particles [100].

The XRD results confirm that the new phyto-nanocarrier was effectively achieved.

#### 2.4.3. Scanning Electron Microscopy (SEM)

Scanning Electron Microscopy (SEM) is a versatile analytical technique used currently to provide insight into surface topography and details on the sample structure.

**Figure 6:**
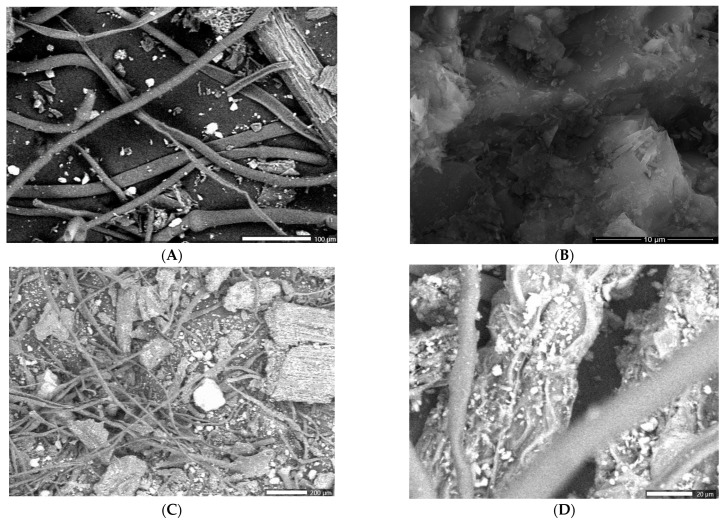
SEM images of ironwort (**A**), clinoptilolite (**B**), and SZ phyto-nanocarrier (**C**,**D**).

Figure 6 shows the SEM micrograph of ironwort, clinoptilolite, and SZ phyto-nanocarrier.

The SEM image of the herb component (Figure 6A) depicts a fibrous surface and porous area alongside several particles of nanometric sizes and irregular shapes. The clinoptilolite micrograph (Figure 6B) exhibits a cluster of cubic and orthogonal nano-sized particles (average size ~30 nm), thus confirming the XDR results. 

The SZ phyto-nanocarrier micrograph (Figure 6C,D) indicates the presence of agglomeration of nanoparticles from herb loaded into clinoptilolite pores and fibrous structures adhering to the surface of the clinoptilolite nanoparticles, probably due to electrostatic interaction. Additionally, it appears that clinoptilolite nanoparticles are loaded into fibrous structures, presumably due to the mechanical forces under employed experimental conditions. On the other hand, SEM indicates a size decrease for both component particles according to the phyto-nanocarrier experimental preparation procedure [101].

#### 2.4.4. Energy Dispersive X-ray (EDX)

**Figure 7:**
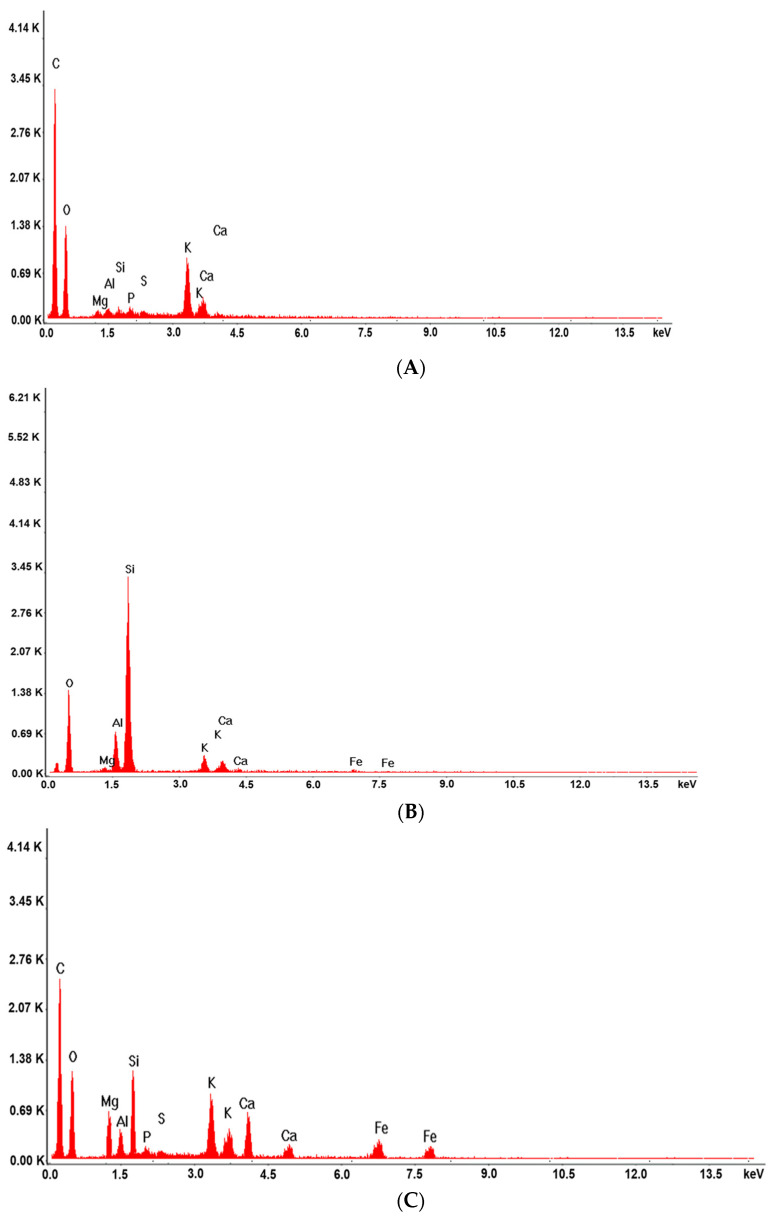
EDX composition of ironwort (**A**), clinoptilolite (**B**), and SZ phyto-nanocarrier (**C**).

The elemental composition of all samples were investigated using (Energy Dispersive X-ray) technique (Figure 7).

According to the EDX spectra (Figure 7B), the clinoptilolite sample is composed of Si (32.31%), Al (7.44%), O (52.43%), K (3.17%), Ca (2.33%), and Fe (0.62%), corroborating the data reported in the literature [102].

The SZ phyto-nanocarrier EDX spectra (Figure 7C) exhibit the peaks corresponding to ironwort (Figure 7A) and clinoptilolite samples, confirming their successful preparation.

### 2.5. Dynamic Light Scattering (DLS)

**Figure 8:**
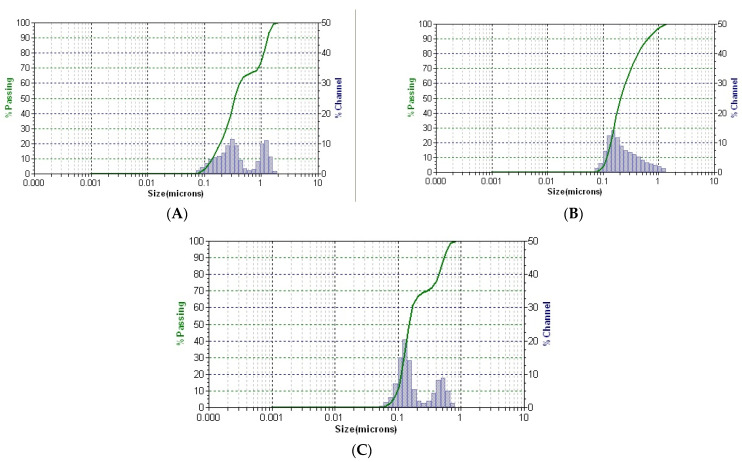
DLS patterns of ironwort (**A**), clinoptilolite (**B**), and SZ phyto-nanocarrier (**C**).

The stability and dynamics of the newly prepared phyto-nanocarrier were evaluated using DLS analysis. The average mean particle size (Table 4) and distribution profile of all samples (Figure 8) were studied using the DLS method.

The dynamic light scattering (DLS) analysis conducted on the ironwort sample (Figure 8A) revealed two distinct peaks attributed to fibrous structures and herb particle presence, with different hydrodynamic diameter values (as indicated in Table 5). Similarly, the DLS pattern of the SZ phyto-nanocarrier (Figure 8C) also exhibited two distinct peaks, which suggested the presence of herb particles (with a diameter size of 130 nm) and clinoptilolite particles (measuring approximately 488 nm). Remarkably, the average diameter of herb particles in the phyto-carrier was significantly lower than that in the herb sample. This difference could be attributed to the experimental procedure employed for phyto-nanocarrier preparation that perhaps led to changes in surface electric charge, which consequently influenced the sample dispersibility and hydrodynamic diameter. Furthermore, the change in size of the clinoptilolite particles from the composition of the phyto-nanocarrier relative to the initial clinoptilolite sample indicated the loading of the pores with plant particles, confirmed by the SEM analysis results. Additionally, Figure 8C indicated that both components were well-dispersed into a narrow range, indicating that the SZ phyto-nanocarrier exhibited high stability. Overall, the DLS analysis provided valuable insights into the properties of the samples, which can be used to devise better experimental protocols for future studies.

### 2.6. Thermal Behaviour Study

**Figure 9:**
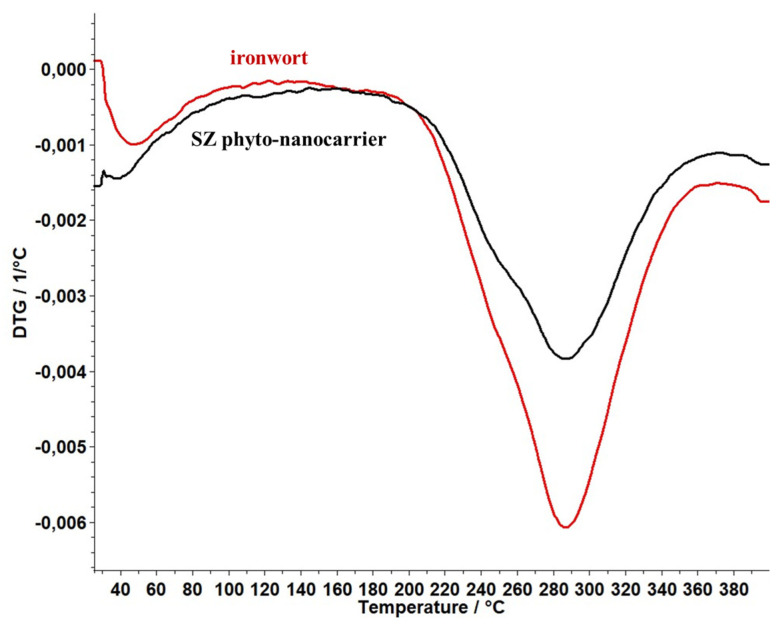
DTG thermograms ironwort sample and SZ phyto-nanocarrier.

**Figure 10:**
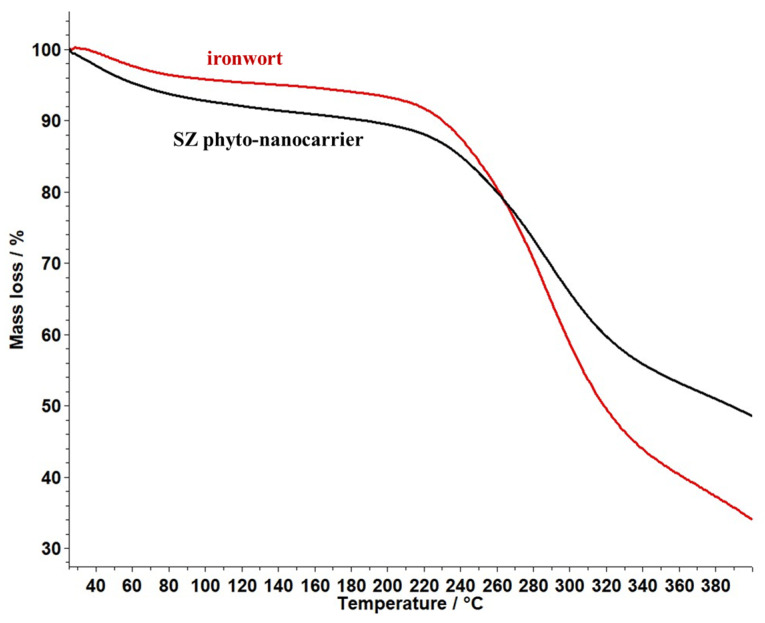
TG thermograms of ironwort sample and SZ phyto-nanocarrier.

The thermogravimetric analysis (TG) and differential thermogravimetric analysis (DTG) methods were used to investigate the physical and chemical changes of ironwort and SZ phyto-nanocarrier as a function of temperature. The results are shown in Figure 9 and Figure 10.

The profile of the thermogravimetric curve (Figure 10) indicates the presence of an endothermic process in the case of both investigated compounds.

According to Figure 9, ironwort displays one important weight loss (53%) in the temperature range of 200–367 °C associated with moisture loss, decarboxylation of volatile compounds, decomposition of phenolics, amino acids, carbohydrates, and polysaccharides [103,104].

The data for the SZ phyto-nanocarrier (Figure 9) exhibited a weight loss (35%) in the temperature range of 200–355 °C attributed to herb biomolecules and, thus, an improvement in thermal stability compared to the ironwort sample.

### 2.7. Screening of Antioxidant Activity and Total Polyphenolic Contents

Considering the great diversity of biomolecules in the chemical composition of medicinal plants, the selection of assays for the antioxidant activity evaluation is required. The most common are chemical or biochemical methods for the total antioxidant activity potential directly estimated from plant matrices [105].

Among the most frequently used are the in vitro tests, divided into two large categories: methods based on the transfer of a hydrogen atom (HAT) or electron transfer (ET). For complex matrices, the most fashionable are the in vitro, non-competitive, non-enzymatic assays for the estimation of antioxidant activity based such as CUPRAC, DPPH, FRAP, ABTS, ABTS/TEAC, or others [106,107,108,109]. In view of the aforementioned grounds, selectivity, accuracy, low cost, and speed are the main criteria for selecting a specific assay [106,107,108,109].

The biological properties of the new phyto-nanocarriers are due to the conjugate and synergic action of the multiple phytoconstituents from the ironwort sample, which are complementary to the carrier (clinoptilolite). Hence, three distinct assays (DPPH, Total polyphenolic contents (Folin-Ciocalteu), and phosphomolybdate (total antioxidant capacity)) were chosen to obtain an accurate insight into the phyto-carrier antioxidant potential.

#### 2.7.1. Total Polyphenolic Contents (TPCs) Assay—Folin Ciocalteu 

The Folin–Ciocalteu assay is a recognizable in vitro method for polyphenol evaluation from plant extracts or other natural product derivatives.

**Figure 11:**
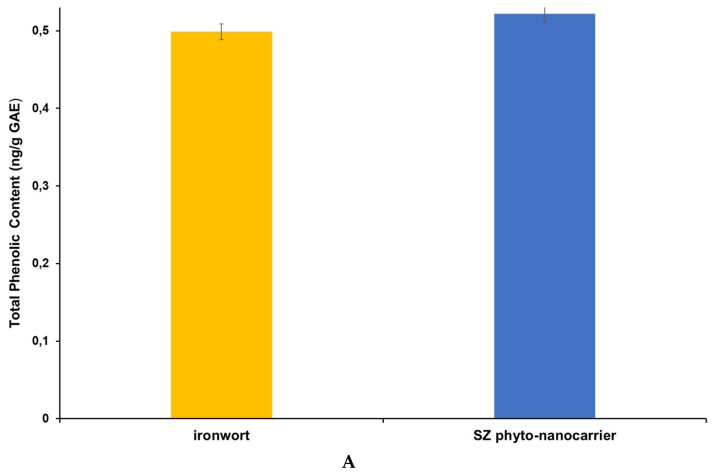

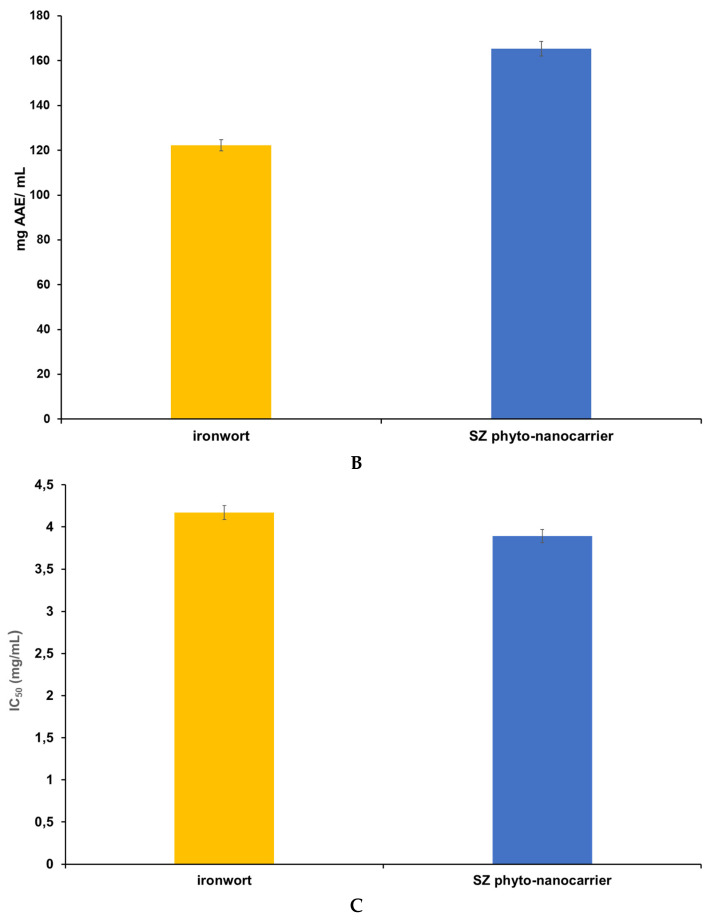
Graphic representation of Total Phenolic (**A**), Total Antioxidant Capacity (**B**), and DPPH (**C**) results.

The results of the TPCs assay obtained for the new phyto-nanocarrier and its components are presented in Table 5 and Figure 11A.

According to the obtained data, a slight increase (about 4.65%) of total polyphenolic content of the SZ phyto-nanocarrier than the ironwort sample, indicating a synergistic action of clinoptilolite [11,12,13,14].

#### 2.7.2. Phosphomolybdate Assay (Total Antioxidant Capacity)

Phosphomolybdate (total antioxidant capacity) is a classical, simple, and accurate technique used for total antioxidant capacity quantitative evaluation from a highly complex mixture of bioactive compounds [110].

Total antioxidant capacity was determined for SZ phyto-nanocarrier, and the data are shown in Table 5 and Figure 11B.

Based on the obtained results, the SZ-phyto-carrier phyto-nanocarrier system has higher antioxidant activity (about 35.3%) than that of the herb sample, suggesting that, under the employed experimental conditions, loading the plant in the clinoptilolite pores induces a favorable effect on antioxidant potential.

#### 2.7.3. DPPH (1,1-diphenyl-2-picrylhydrazyl) Free-Radical-Scavenging Assay

2,2-diphenyl-1-picrylhydrazyl (DPPH) assay is a straightforward, specific, sensitive, fast technique for the free radical DPPH scavenging capacity assessment in various heterogenous mixtures [109].

Results of DDPH for SZ phyto-nanocarrier and herb component are presented in Table 5 and Figure 11C.

According to the data, and given that lower IC_50_ values correspond to higher antioxidant activity, the SZ phyto-nanocarrier exhibits a slight increase (up to 7.2%) compared to the ironwort sample, might be due to the biomolecules loaded in clinoptilolite pores, which is in good agreement with the literature [11,12,13,14].

The collective results of the antioxidant assays used in this study indicate that the new phyto-nanocarrier prepared has a very high antioxidant activity compared to the plant sample, considering the influence of the reaction parameters (molar ratio, solvent polarity, pH, temperature) such as and the modification of the structural properties (size particles, specific surface, etc.) occurring as a result of the employed experimental procedure [111,112]. Various studies reported that loading the zeolite pores with phytochemicals enhances in vivo biocompatibility and stability, as well as prolonged controlled release [18,19].

### 2.8. In Vitro Dissolution Assay

In vitro, dissolution testing is extensively used as a significant technique to evaluate in vivo performance in the digestive tract and, implicitly, their bioavailability and therapeutic efficiency. 

Herbal preparations contain numerous phytoconstituents (belonging to distinct chemical categories) in various proportions depending on different biotic and abiotic parameters (including the selected extraction procedures), which determines variations in adsorption properties and pharmacological effectiveness [113,114,115,116,117,118]. Consequently, in vivo, biopharmaceutical performance mimic and prediction for natural products is far more challenging than single-component synthetic drugs [119,120,121].

The physiological pH in the human digestive tract varies from 1.2 to 7.8. Hence, the appropriate dissolution environment provides valuable information on absorption properties. Commonly, in aqueous media, small amounts of various surfactant types are added to promote the solubility of some phytoconstituents.

The dissolution profile of ironwort and SZ phyto-nanocarrier at two different pH values (pH 4 and pH 7) were investigated as a function of time [117,118].

**Figure 12:**
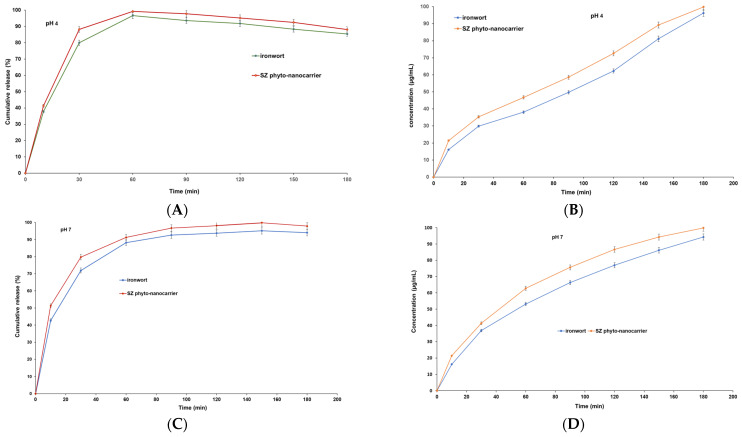
Dissolution profile of ironwort and SZ phyto-nanocarrier.

To that end, the relationship between pH and dissolution rates is shown in Figure 12.

The results indicate that both samples had increased dissolution time in an acidic environment (pH 4) over time. Although both samples exhibited a relatively similar dissolution profile, it can be observed that the SZ phyto-nanocarrier performed better, displaying a dissolution of about 25% until 120 min, compared to the ironwort sample. Subsequently, this difference decreased to approximately 15% (Figure 12). Both samples demonstrated a high degree of rapid release within 30 min, about 75% for ironwort and about 85% for SZ phyto-nanocarrier, reaching a maximum of 96.37% for ironwort and 99.76% for SZ phyto-nanocarrier at 60 min (Figure 12A,C). These results are in good agreement with the requirements imposed by international regulations [116].

The dissolution rate showed a notable shift with the pH modification. At pH 7, the release rate was still fast, about 72% for ironwort and 80% for SZ phyto-nanocarrier, but slightly lower than at pH 4 (Figure 12A,C). The results indicate that the maximum release was reached at 150 min for both samples, 94% for ironwort and 99% for SZ phyto-nanocarrier (Figure 12A,C).

Recent literature results have reported that the bioavailability of a drug depends on various physical characteristics of drug delivery, such as particle size, shape, and long-range structural (atomic-scale) order [117,118].

Therefore, the enhanced dissolution rate of the SZ phyto-nanocarrier can be attributed to the reduction in particle size and increased specific surface area under the employed experimental conditions. Furthermore, the variation of the dissolution speed with the pH value suggests the formation of electrostatic or van der Waals interactions. In an acid medium (pH = 4), both investigated compounds (ironwort and SZ phyto-nanocarrier) demonstrated a higher dissolution rate than the value obtained at pH = 7, indicating that it is most suitable for adsorption. These findings provide valuable insights into the potential applications of SZ phyto-nanocarrier in the pharmaceutical and nutraceutical industries.

## 3. Materials and Methods

### 3.1. Reagents and materials

All reagents used in this study were of analytical grade, acquired from commercial suppliers, and used without further purification. Methanol, ethanol, dichloromethane, chloroform, gallic acid, ascorbic acid, 2,2-diphenyl-1-picrylhydrazyl, sodium phosphate, potassium chloride (99% were also purchased from), anhydrous sodium carbonate, potassium persulfate, ammonium molybdate, and Folin–Ciocalteu phenol reagent (2 N) were purchased from Sigma-Aldrich (München, Germany). Buffer solution pH 7 (di-sodium hydrogen phosphate/potassium dihydrogen phosphate), Buffer solution pH 4 (citric acid/sodium hydroxide/hydrogen chloride) (Merck Millipore (Darmstadt, Germany). Ultrapure water was used in all experiments.

Clinoptilolite samples (Baia Mare, Romania, were offered by the University Politehnica Timisoara (Timisoara, Romania). It was micronized using a planetary mill Fritsch Pulverisette mill (Idar-Oberstein, Germany) (500 rpm for 20 min at 23 °C) and then sieved through several ASTM sieves. The present study made use only of particle size within the range of 1.3–3.0 µm. Subsequently, the clinoptilolite sample was washed with ultrapure water, dried at 100 °C, and then stored in a desiccator [95].

*Sideritis scardica Griseb*. (ironwort) samples (dried stems, leaves, and flowers) were provided by the University of Medicine and Pharmacy Craiova, Romania.

### 3.2. Plant Samples’ Preparation for Chemical Screening

The ironwort samples were milled using a planetary mill, sieved to obtain particle diameters ranging from 0.25–0.30 mm, and then stored until further use. The ultrasonic-assisted extraction was carried out using (1.5 g dried plant sample: 20 mL solvent) a methanol/chloroform mixture (1:1, *v*/*v*) at 35 °C and 60 Hz for 30 min (Elmasonic, Singen, Germany). Then, the solvent mixture was removed using a rotary evaporator (Rotavapor; BÜCHI, Flawil, Switzerland) at 30 °C. The residue obtained was dissolved with MeOH (15 mL), centrifuged, and then filtered (0.20 μm) and stored at −30 °C. All experiments were prepared in triplicate.

#### 3.2.1. GC-MS Analysis (EZ: Faast GC-MS Free Amino Acids Kit)

Gas chromatography was carried out using a GCMS-QP2020NX Shimadzu apparatus (Shimadz, Kyoto, Japan) with a ZB-5MS capillary column (30 m × 0.25 mm id × 0.25 µm) (Phenomenex, Torrance, CA, USA), helium (flow rate of 1 mL/min).

##### GC-MS Separation Conditions

The oven temperature was increased from 50 °C (kept for 2 min) to 300 °C (rate of 5 °C/min, kept for 5 min). The temperature of the injector was 300 °C, and the temperature at the interface was 215 °C. The mass of the compounds was registered at a 70 eV ionization energy. The mass spectrometer was source-heated at 220 °C, and the MS Quad was heated at 150 °C. The results obtained are shown in Table 1.

#### 3.2.2. Mass Spectrometry

The MS experiments were performed using an EIS-QTOF-MS (Bruker Daltonics, Bremen, Germany). The mass spectra were acquired in the positive ion mode in a mass range of 70–3000 *m*/*z*. The scan speed was 2.2 scans/s, the collision energy was 10–85 eV, and the temperature of the source block was 80 °C [38,51,79,99].

Compounds were identified based on their mass spectra, which were compared to the NIST 3.0 database mass spectra library database (USA National Institute of Science and Technology software) (NIST, Gaithersburg, MD, USA) and literature review. The biomolecules identified are presented in Table 2.

### 3.3. SZ Phyto-Nanocarrier

To prepare the SZ phyto-nanocarrier, the ironwort sample (dried herb) and clinoptilolite were added (1:2.5 mass ratio), then ground, and homogenized for 10 min using planetary mill Fritsch Pulverisette mill. Each experiment was repeated three times.

### 3.4. Characterization of Phyto-Nanocarrier and Its Components

#### 3.4.1. Fourier Transform Infrared (FT-IR) Spectroscopy

FT-IR spectra of SZ phyto-nanocarrier, ironwort, and clinoptilolite were recorded (range of 400–4000 cm^−1^, resolution of 4 cm^−1^) on a Fourier transform infrared spectrometer (Shimadzu AIM-9000 with ATR devices).

#### 3.4.2. XRD Spectroscopy

The phase composition of the phyto-nanocarrier and each component were investigated using a Bruker AXS D8-Advance X-ray diffractometer (Bruker AXS GmbH, Karlsruhe, Germany) equipped with a rotating sample stage, Anton Paar TTK low-temperature cell (−180–450 °C), high-vacuum, inert atmosphere, relative humidity control. The average crystallite size and the phase content were determined using the whole-pattern profile-fitting method (WPPF).

#### 3.4.3. Scanning Electron Microscopy (SEM)

Morpho-structural analysis was conducted on a SEM-EDS system (JEOL JSM-IT200 Field Emission, Nieuw-Vennep, The Netherlands) equipped with a high-resolution electron gun and energy-dispersive X-ray spectrometer (EDS).

#### 3.4.4. Dynamic Light Scattering (DLS) Particle Size Distribution Analysis

The DLS analysis was performed on a scattering angle of 172° at room temperature (23 °C) using a Microtrac/Nanotrac 252 (Montgomeryville, PA, USA) instrument. Each experiment was repeated three times.

### 3.5. Thermal Analysis

The thermal stability of the SZ phyto-nanocarrier and ironwort sample was studied in a dynamic air atmosphere (20 mL/min, synthetic air) at the temperature range (25 and 400 °C), with a 10°/min heating rate) using a Thermal Analyzer produced by Mettler Toledo, model TGA/DSC3^+^ STARe System. The DSC analysis was performed in an air atmosphere (50 mL/min) with a temperature range (25–400 °C) on a DSC 3+ Mettler Toledo.

### 3.6. Antioxidant Activity and Total Phenolic Content

The antioxidant potential of SZ phyto-nanocarrier and its components were estimated employing three different assays: total phenolic content (Folin–Ciocalteu) assay, 2,2-diphenyl-1-picrylhydrazyl, (DPPH) radical scavenging assay and phosphomolybdate assay (total antioxidant capacity). The experimental procedure was the following: Round-bottom flasks (50 mL) containing 0.20 (±0.001) g solid sample to which 10 mL ethanol (70%) was added. Subsequently, the mixture was subjected to sonication extraction for 30 min, 200 rpm, at 45 °C. Extracted solutions were centrifuged at 3500 rpm for 10 min, and then the supernatant was collected for use in the selected antioxidant assays for this study.

#### 3.6.1. Total Polyphenol Content Using the Folin–Ciocalteu Method

The total phenolic content in SZ phyto-nanocarrier and its components were determined using a UV-VIS spectrophotometry (DLAB SP-UV1000, Penjuru, Singapore), according to the Folin–Ciocalteu experimental procedure adapted from the literature [122].

The sample extract concentrations were calculated based on the linear equation obtained from the standard curve (y = 0.0028x + 0.0264 and the correlation coefficient (R^2^ = 0.9967).

#### 3.6.2. DPPH Radical Scavenging Assay

The antioxidant activities of SZ phyto-nanocarrier and ironwort samples were comparatively investigated using a DPPH (2,2-diphenyl-1-picrylhydrazyl) free radical elimination assay. The extract samples were prepared at different concentrations in the range of 0.020 and 20 mg/mL. Then, 1.5 mL of DPPH working solution was transferred to all sample extracts (10 μL), mixed, and left in the dark for 30 min. All analyses were carried out in triplicates, and absorbance was recorded at 510 nm. The results were used to determine the inhibition percentage (Inh%) according to Equation (1).

Inh% = (A_0_ − A_l_)/A_0_ × 10 (1)

The IC_50_ (half-maximal inhibitory concentration) values were obtained from the inhibition percentage using the equation from the calibration curve generated for each sample.

#### 3.6.3. Phosphomolybdate Assay (Total Antioxidant Capacity)

The total antioxidant capacity assay of SZ phyto-nanocarrier and ironwort sample was carried out according to phosphomolybdenum, a procedure described in our earlier publication [111]. The results are presented as μg/mL of ascorbic acid equivalents (AAE).

### 3.7. In Vitro Dissolution Test

Dissolution profiles of ironwort (0.5 g ± 0.045) and SZ phyto-nanocarrier (0.5 g ± 0.019) were determined using a 708-DS Dissolution -Agilent Technologies (Santa Clara, CA, USA) at 37 ± 0.5 °C, 100 rpm in 600 mL of two buffers of distinct pH value: pH 4 (simulated the stomach fluids) and pH 7 (simulated the intestinal fluids) and sink conditions were maintained throughout the dissolution rate tests [123].

The evaluation of dissolution profiles was carried out at 15, 30, 45, 60, 90, and 120 min.

A total of 15 mL of an aliquot of the dissolution medium was automatically collected at the set time interval and filtrated (0.45 μm). The cumulative drug released against time was determined using a UV-Vis Perkin-Elmer Lambda 35 (Perkin Elmer, Waltham, MA, USA) [123].

Triplicate samples were analyzed at each time. The mean value of six samples and a standard deviation were calculated [124].

#### Preparation of the Curves of the Concentrations for the Compound Dissolution Profile

Different solution concentrations (in the range of 0.001 and 0.25 mg/mL) were prepared from each compound (ironwort and SZ phyto-nanocarrier, respectively). Calibration curves were plotted for ironwort and SZ phyto-nanocarrier. The amount of each compound released was obtained from the standard curve of the concentration versus its absorbency. The correlation coefficients, at pH = 4 were: R^2^ = 0.9987 (ironwort) and R^2^ = 0.9978 (SZ phyto-nanocarrier); at pH = 7, R^2^ = 0.9989 (ironwort) and R^2^ = 0.9973 (SZ phyto-nanocarrier) demonstrate the good linear relationship of the data.

The compound release was calculated according to the following equation [125]

(2) $$CDR\left(\%\right)=\frac{amount\;of\;released\;compound\;at\;time\;n\;(g)}{amount\;compound\;used\;as\;raw\;materials\;(g)}\times 100$$

### 3.8. Statistical Analysis

Each experimental set was performed in triplicate, using one-way analysis of variance (ANOVA) without replication with Scheffe’s post hoc test comparison; *p* < 0.05 was taken as statistically significant. Data are presented as the means ± SD.

## 4. Conclusions

This study presents the preparation and a comprehensive evaluation conducted to assess the potential of a newly developed nanocarrier derived from *Sideritis scardica Griseb* and clinoptilolite. The unique features of the new nanocarrier were confirmed through various analytical methods (FT-IR, XRD, SEM-EDX, DLS).

Accordingly, the reduction in particle size and specific surface area increases due to loading the herb into clinoptilolite pores, allowing better thermal stability and an increase in antioxidant potential and dissolution rate compared to the herb sample. Based on the results, the nanocarrier shows great promise as a candidate for drug delivery systems. However, further research is needed to explore its potential biomedical applications.

## Figures and Tables

**Table 1:** GC-MS amino acids identification from *Sideritis scardica* sample.

Proposed Structure	Abbreviation	SIM (Selected Ion Monitoring)
alanine	Ala	130, 70
glycine	Gly	116, 74
leucine	Leu	172, 86
isoleucine	Ile	172, 130
threonine	Thr	160, 101
serine	Ser	146, 203
proline	Pro	156, 243
asparagine	Asn	155, 69
aspartic acid	Asp	216, 130
methionine	Met	203, 277
phenylalanine	Phe	206, 190
glutamic acid	Glu	230, 170
lysine	Lys	170, 128
histidine	His	282, 168

**Table 2:** Biomolecules identified in *Sideritis scardica* sample through MS analysis.

No	Detected *m*/*z*	Theoretic *m*/*z*	Formula	Tentative of Identification	Category	Ref.
1	76.08	75.07	C_2_H_5_NO_2_	glycine	amino acids	[37,38,51]
2	90.11	89.09	C_3_H_7_NO_2_	alanine		[37]
3	106.07	105.09	C_3_H_7_NO_3_	serine		[37]
4	116.11	115.13	C_5_H_9_NO_2_	proline		[37]
5	120.13	119.12	C_4_H_9_NO_3_	threonine		[37]
6	132.15	131.17	C_6_H_13_NO_2_	isoleucine		[37]
7	133.13	132.12	C_4_H_8_N_2_O_3_	asparagine		[37]
8	134.11	133.10	C_4_H_7_NO_4_	aspartic acid		[37]
9	147.18	146.19	C_6_H_14_N_2_O_2_	lysine		[37,38,51]
10	148.14	147.13	C_5_H_9_NO_4_	glutamic acid		[37]
11	149.23	149.21	C_5_H_11_NO_2_S	methionine		[37,38,51]
12	156.17	155.15	C_6_H_9_N_3_O_2_	histidine		[37,38,51]
13	166.17	165.19	C_9_H_11_NO_2_	phenylalanine		[37]
14	271.25	270.24	C_15_H_10_O_5_	apigenin	flavonoids	[5,45,46,48]
15	285.27	284.26	C_16_H_12_O_5_	genkwanin		[41]
16	287.23	286.24	C_15_H_10_O_6_	luteolin		[45,46,48]
17	301.27	300.26	C_16_H_12_O_6_	chrysoeriol		[5,45,48]
18	303.25	302.23	C_15_H_10_O_7_	hypolaetin		[48]
19	315.28	314.29	C_17_H_14_O_6_	cirsimaritin		[41,49]
20	317.25	316.26	C_16_H_12_O_7_	nepetin		[49]
21	329.28	328.30	C_18_H_16_O_6_	salvigenin		[49]
22	343.35	342.34	C_16_H_22_O_8_	coniferin		[41]
23	345.21	344.22	C_18_H_16_O_7_	eupatorin		[41]
24	651.61	650.60	C_30_H_34_O_16_	tremasperin		[41]
25	165.17	164.16	C_9_H_8_O_3_	*p*-coumaric acid	phenolic acids	[45]
26	169.14	168.15	C_8_H_8_O_4_	vanillic acid		[45]
27	171.13	170.12	C_7_H_6_O_5_	gallic acid		[45]
28	181.15	180.16	C_9_H_8_O_4_	caffeic acid		[45]
29	193.15	192.17	C_7_H_12_O_6_	quinic acid		[48]
30	195.17	194.18	C_10_H_10_O_4_	ferulic acid		[45]
31	199.15	198.17	C_9_H_10_O_5_	syringic acid		[45]
32	355.33	354.31	C_16_H_18_O_9_	chlorogenic acid		[5,45]
33	135.21	134.22	C_10_H_14_	o-cymene	terpenoids	[42]
34	137.24	136.23	C_10_H_16_	α-thujene		[43,44]
35	149.19	148.20	C_10_H_12_O	anethone		[43]
36	151.21	150.22	C_10_H_14_O	tymol		[43]
37	153.25	152.23	C_10_H_16_O	camphor		[43]
38	154.25	154.25	C_10_H_18_O	borneol		[43]
39	157.25	156.26	C_10_H_20_O	menthol		[43]
40	191.27	190.28	C_13_H_18_O	damascenone		[47]
41	193.31	192.30	C_13_H_20_O	beta-damascone		[43]
42	199.31	198.30	C_15_H_18_	cadalene		[43]
43	202.34	202.33	C_15_H_22_	α-curcumene		[46]
44	205.35	204.35	C_15_H_24_	humulene		[43]
45	219.31	218.33	C_15_H_22_O	germacrone		[48]
46	221.34	220.35	C_15_H_24_O	spatulenol		[40,43]
47	223.36	222.37	C_15_H_26_O	valeranone		[43]
48	297.49	296.50	C_20_H_40_O	phytol		[43]
49	305.49	304.50	C_20_H_32_O_2_	sideridiol		[45]
50	321.49	320.50	C_20_H_32_O_3_	sideroxol		[45,48]
51	323.51	322.50	C_20_H_34_O_3_	andalusol		[49]
52	333.41	332.40	C_20_H_28_O_4_	carnosic acid		[41]
53	347.49	346.50	C_22_H_34_O_3_	siderol		[45,48]
54	363.51	362.50	C_22_H_34_O_4_	eubol		[45,48]
55	463.41	462.40	C_20_H_30_O_12_	forsythoside	phenylethanoid glycosides	[48]
56	625.59	624.60	C_29_H_36_O_15_	verbascoside		[5]
57	639.58	638.60	C_30_H_38_O_15_	leucosceptoside A		[48,50]
58	653.58	652.60	C_31_H_40_O_15_	martynoside		[48,50]
59	669.62	668.60	C_31_H_40_O_16_	verbascoside		[48]
60	757.71	756.70	C_34_H_44_O_19_	lavandulifolioside		[48,50]
61	771.68	770.70	C_35_H_46_O_19_	alyssonoside		[48,50]
62	787.70	786.70	C_35_H_46_O_20_	echinacoside		[48,50]
63	375.33	374.34	C_16_H_22_O_10_	geniposidic acid	iridoids	[41]
64	449.39	448.40	C_19_H_28_O_12_	barlerin		[41]
65	525.51	524.50	C_21_H_32_O_15_	melittoside		[42]
66	173.27	172.26	C_10_H_20_O_2_	capric acid	fatty acids	[43]
67	201.33	200.32	C_12_H_24_O_2_	lauric acid		[43]
68	257.41	256.42	C_16_H_32_O_2_	palmitic acid		[43]
69	281.39	280.40	C_18_H_32_O_2_	linoleic acid		[43]
70	283.51	282.50	C_18_H_34_O_2_	oleic acid		[43,46]
71	401.69	400.70	C_28_H_48_O	campesterol	sterols	[48]
72	413.69	412.70	C_29_H_48_O	stigmasterol		[48]
73	415.71	414.70	C_29_H_50_O	β sitosterol		[48]
74	245.21	244.20	C_9_H_12_N_2_O_6_	uridine	nucleosides	[41]
75	284.25	283.24	C_10_H_13_N_5_O_5_	guanisine	nucleosides	[41]
76	103.15	102.17	C_6_H_14_O	hexanol	alcohols	[40]
77	129.19	128.21	C_8_H_16_O	oct-1-en-3-ol		[40]
78	271.49	270.50	C_18_H_38_O	2-octadecanol		[43]
79	207.27	206.28	C_13_H_18_O_2_	hexylbenzoate	esters	[39]
80	213.25	212.24	C_14_H_12_O_2_	benzylbenzoate		[39]
81	295.51	294.50	C_19_H_34_O_2_	methyl lineoleate		[43]
82	313.51	312.50	C_20_H_40_O_2_	stearyl acetate		[39]
83	143.27	142.28	C_10_H_22_	decane	hydrocarbons	[43]
84	325.59	324.60	C_23_H_48_	tricosane		[43]
85	353.71	352.70	C_25_H_52_	pentacosane		[43]
86	121.17	120.15	C_8_H_8_O	acetophenone	ketones	[40]
87	165.25	164.24	C_11_H_16_O	cis-jasmone		[46]
88	123.11	122.12	C_7_H_6_O_2_	benzoic acid	organic acids	[40]
89	135.07	134.09	C_4_H_6_O_5_	malic acid		[41]
90	151.07	150.09	C_4_H_6_O_6_	tartaric acid		[41]
91	201.21	200.23	C_10_H_16_O_4_	camphoric acid		[42]

**Table 3:** The characteristic absorption bands attributed to the phytoconstituents from *Sideritis scardica*.

Phytoconstituent	Wavenumber (cm^−1^)	Ref.
terpenoids	2940, 1745, 1700, 1650, 810	[78,79]
phenylethanoid glycosides	3390, 2853, 2925, 1720, 1700, 1628, 1602, 1520, 1454,1385, 1280	[80,81,82]
fatty acids	3600, 3020–3010, 2960, 2925, 2875, 2850, 2560, 1702, 1350, 1245, 724	[83,84,85]
flavonoids	4000–3124, 3140–2980, 1655, 1644, 1620, 1585, 1496, 1465, 1415, 1370, 1275, 1080, 770, 535	[86]
phenolic acids	1800–1650, 1730, 1640, 1625, 1518, 1440, 1412, 1365, 1310, 1260, 1165, 1090, 945, 805	[79,87]
iridoids	1740–1458, 1377, 1221–914	[88]
phytosterols	3425, 3350, 2935, 2832, 1752, 1642, 1465, 1385, 1190, 1065, 990, 950, 880, 741	[89,90]
amino acids	3400; 3330–3130; 2985, 2360–2530, 2130; 1725–1755 1690, 1675, 1665, 1650, 1645, 1630, 1625, 1610, 1500–1600	[91]
nucleoside	3350, 3105, 2925, 2800, 1670, 1470, 1395, 1270, 1210, 1140. 1095, 1055, 980, 905, 830, 770, 570, 450	[92]

**Table 4:** The DLS mean hydrodynamic diameter values of the SZ phyto-nanocarrier and both components.

Sample	Diameters (µm)	Width (µm)
ironwort	1.1710	0.4540
	0.2609	0.2383
clinoptilolite	0.2052	0.3530
SZ phyto-nanocarrier	0.4880	0.2170
	0.1299	0.0637

**Table 5:** The results of phenolic contents, total antioxidant capacity, and IC_50_ values for ironwort and SZ phyto-nanocarrier.

Sample Name	Total Phenolic Content (mg/g GAE)	mg AAE/mL	IC_50_ (mg/mL)
ironwort	0.4988 ± 0.072	122.23 ± 0.015	4.17 ± 0.019
SZ phyto-nanocarrier	0.5219 ± 0.010	165.37 ± 0.023	3.89 ± 0.034

## Data Availability

Data is contained within the article.
